# Neuroinflammation is a Key Player in the Cognitive Fate of Individuals Who Harbor Alzheimer Disease Neuropathologic Changes

**DOI:** 10.1002/alz70855_107046

**Published:** 2025-12-24

**Authors:** Sunny Kumar, Ibai Diez, Ana Claudia Amaral, Cinthya Aguero, Charles Z Klein, Teresa Gomez‐Isla

**Affiliations:** ^1^ Harvard Medical School, Boston, MA, USA; ^2^ Massachusetts General Hospital, Boston, MA, USA; ^3^ MassGeneral Institute for Neurodegenerative Disease, Charlestown, MA, USA; ^4^ Massachusetts General Hospital, Harvard Medical School, Boston, MA, USA; ^5^ Mass General Institute for Neurodegenerative Disease, Charlestown, MA, USA

## Abstract

**Background:**

Some individuals show no cognitive decline during life despite presence in their brains of Alzheimer disease neuropathologic changes (ADNC) (e.g. amyloid plaques and tangles). Understanding the molecular mechanisms underlying resilience to ADNC could help develop new biomarkers and identify novel therapeutic targets.

**Method:**

We compared brain changes and gene expression profiles of resilient and demented AD brains exhibiting equivalent loads of ADNC at autopsy. We performed quantitative neuropathological assessments on post‐mortem brain samples containing superior temporal sulcus and analyzed gene expression profiles on available RNA‐seq data from dorsolateral prefrontal cortex of ROSMAP participants from the AMP‐AD Knowledge Portal (Synapse IDs: syn22283382; syn2230160). We included 28 cases that fulfilled neuropathologic criteria for high likelihood of AD (14 demented and 14 cognitively normal 'resilient') and 21 that fulfilled criteria for low probability of AD (cognitively normal ‘control’) at autopsy. Gene expression analyses were conducted using DESeq2 R package. Age, gender, sequencing batch, and post‐mortem interval were accounted for in statistical analyses. A Wald test was employed to identify differentially expressed genes (DEGs) between demented AD and resilient.

**Result:**

Demented AD and resilient brains had equivalent loads of amyloid plaques and number of neurofibrillary tangles, but demented AD had significantly higher tau neuropil thread burdens and increased pTau levels in synaptosome‐enriched fractions than resilient. Demented AD also showed increased burdens of activated astrocytes (GAFP+) and microglia (CD68+) compared to resilient. The Gene ontology functional analyses revealed significant reduction in apoptotic process, neuroinflammation/response to cytokines, and protein phosphorylation processes in resilient compared to demented AD. Pathway analyses with DEGs revealed downregulation of TNF, JAK‐STAT, and NF‐κB signaling pathways in resilient compared to demented AD. TNF and NF‐kB pathways shared a common downregulation of CXCL1 and CXCL2 gene expression in resilient compared to demented AD cases.

**Conclusion:**

Based on the above findings, we propose that aberrant accumulation of pathological tau species in neurites and synapses may instigate a pro‐inflammatory reaction of glial cells that involves activation of TNF and NF‐κB signaling pathways and could determine the opposite cognitive fate (dementia vs. preserved cognition) of individuals who harbor ADNC as assessed at autopsy.

